# Oral health-related quality of life in patients aged 8 to 19 years with cleft lip and palate: a systematic review and meta-analysis

**DOI:** 10.1186/s12903-023-03382-4

**Published:** 2023-09-16

**Authors:** Augusto Garcia de Oliveira Júnior, Erik Montagna, Victor Zaia, Caio Parente Barbosa, Bianca Bianco

**Affiliations:** 1 Postgraduation Program in Health Sciences, Faculdade de Medicina do ABC/Centro Universitário FMABC, Santo André, Brazil; 2 Discipline of Sexual and Reproductive Health, and Populational Genetics, Department of Collective Health, Faculdade de Medicina do ABC/Centro Universitário FMABC, Av. Lauro Gomes, 2000, Santo André, CEP 09060-870 SP Brazil

**Keywords:** Cleft lip and palate, Oral health-related quality of life, Psychometry, Child and adolescence

## Abstract

**Background:**

Cleft lip and palate (CLP) is the most common facial birth defect worldwide and causes morphological, aesthetic, and functional problems with psychosocial implications for an individual’s life and well-being. The present systematic review and meta-analysis assessed whether the treatment of CLP impacts the oral health-related quality of life (OHRQoL) in children and adolescents in comparison to healthy controls.

**Methods:**

We searched MEDLINE/PubMed, EMBASE, and PsycINFO databases using terms related to CLP, and included articles until August 2023. Observational comparison studies that assessed OHRQoL in non-syndromic CLP patients aged 8–19 years with validated scales designed to such aim or scales capable to identify aspects related to oral health compared to healthy controls were included. We used the ROBINS-I tool for risk of bias assessment. A meta-analysis of continuous variables was performed using inverse variance for pooling estimates, Standardized Mean Difference (SMD) as a summary measure, with random effects model. Heterogeneity was estimated by the I^2^ statistics. Sensitivity analyses included subgrouping based on the scale, risk of bias and scale domains. Meta-regression was performed under a mixed-effects model considering the variables type of scale, scale domains and risk of bias.

**Results:**

Fourteen studies were included comprising 1,185 patients with CLP and 1,558 healthy controls. The direction of the effect of OHRQoL favoured the healthy group (-0.92; 95% CI:-1,55;-0,10) and I^2^ = 95%. After removing three studies, I^2^ dropped to 80%. Meta-regression showed no influence on risk of bias (p = 0.2240) but influence of scale type (p = 0.0375) and scale domains (p < 0.001). The subgroup analysis indicated that the CPQ and COHIP scales presented very discrepant SMD values, despite pointing to the same effect direction. In contrast, the OHIP scale showed a non-significant difference between cases and controls, with estimates much lower than the other two scales. Results also suggest that OHRQoL associated with oral functionality and social well-being is more influential on outcomes than emotional well-being.

**Conclusion:**

The global OHRQoL is slightly worst in the CLP patients than control group. The difference between OHRQoL was mainly detected through OHIP. The most affected domains are functional, emotional and social.

**Systematic review registration:**

PROSPERO CRD42022336956.

## Background

Cleft lip and palate (CLP) are congenital malformations characterized by gaps or discontinuity of the structures of the lip and/or palate that occur during the embryonic period (up to the 12th week of gestation) and are variable in location and extent. It is the fourth most common congenital anomaly in humans [1], behind congenital clubfoot, syndactyly, polydactyly, and neural tube closure defects [2]. The occurrence of cleft lip with or without cleft palate is 1:1000 live births, and the occurrence of cleft palate alone is approximately 1:2500 live births. The prevalence of CLP is higher in Asians (mainly Japanese) and lower in Afro-descendants than in Caucasians [3]. Men are more often affected by cleft lip with or without cleft palate (2:1), while CLP is more common in women (1:0.5) [4, 5].

CLP can be classified as syndromic or non-syndromic, with 70% being non-syndromic [6]. Approximately 600 syndromes have been associated with CLP [5]. The etiology of CLP is multifactorial and includes genetic causes (variants in the *IRF6*, *VAX1*, and *PAX7* genes); malnutrition; endocrine disorders; infections; trauma; and alcohol consumption; as well as other environmental causes such as smoking, pre-and gestational diabetes, and the use of medications such as corticosteroids and anticonvulsants. Approximately 20% of CLP occurs in consanguineous families [7] and the genetic component is demonstrated by an increased recurrence rate among affected families. The risk of recurrence between affected parents is 3%; when a sibling is affected, it increases to 5%, and if both parent and sibling are affected, there is a risk of recurrence of 14% [8].

Considering the frequency of concomitant abnormalities, early dysmorphological assessment is essential. A comprehensive genetic evaluation should be considered in the presence of additional abnormalities. Not only the facial appearance but also functions such as hearing, phonation, mastication, swallowing, and ventilation are altered by this malformation [9]. Patients with CLP often require multiple medical specialties and must be followed up by a multidisciplinary team, mainly an orthodontist, from the first days of life to early adulthood [10]. Surgical reconstruction of the cleft palate is aimed at restoring the palatal length and function to facilitate the development of intelligible speech. However, many children continue to have clinically apparent speech disturbances even after primary palate repair, and approximately 15% undergo secondary surgery to improve palatal length and competence [11].

CLP results in morphological, aesthetic, and functional problems with psychosocial implications for the life and well-being of individuals, ranging from low self-esteem to the risk of social isolation [10]. Several oral health complications are present, including tooth agenesis and supernumerary and/or malpositioned teeth that cause speech disorders, such as hypernasality, as well as facial changes, such as nose and mouth asymmetry, which also affect an individual’s self-image, social behavior, and adaptation [12].

Previous studies have suggested that facial esthetics are an important aspect of quality of life (QoL) in individuals with repaired CLP and that satisfaction with facial appearance is positively correlated with health-related QoL [13–15]. As oral health is part of general health and is essential for the maintenance of the QoL, the term “oral health-related quality of life” (OHRQoL) has been used to refer to the impact of oral health or diseases on the daily life of individuals [16].

In the long term, treatment for CLP is expected to result in esthetic and functional improvements with a positive impact on speech and occlusion [17], as well as the psychological and social well-being of affected people and their families [18–21].

The years close to adolescence are considered psychologically difficult for populations with and without CLP. Adolescents with CLP must cope with problems associated with facial appearance, the process of the changing body, and the development of romantic relationships, despite dissatisfaction with appearance. Additionally, these adolescents often discuss surgical procedures with their parents and show more behavioral problems related to internalizing and externalizing [12]. Older age and female sex typically have a greater impact on OHRQoL in patients with CLP; however, these findings remain controversial [22]. A recent publication of outcomes related to orthodontic treatment in patients with CLP found that QoL and the use of health resources are the least reported outcomes in the literature [22].

Inspired by these findings, the present study aimed to assess and compare the OHRQoL in children and adolescents with and without CLP only with specific psychometric scales.

## Methods

### Systematic review and meta-analysis

A systematic review was conducted according to the Preferred Reporting Items for Systematic Reviews and Meta-Analyses (PRISMA) statement [23]. This study was registered in International Prospective Register of Systematic Reviews (PROSPERO CRD42022336956).

The study question is whether the treatment of cleft lip and palate impacts the oral-related quality of life of patients from age 8–19 years who underwent surgical treatment in comparison to individuals of the same age without the condition.

### Search strategy and selection criteria

The MEDLINE/PubMed, EMBASE, PsycINFO, SciELO, Scopus and Web Of Science databases were searched. To identify the structured question, instead of PICOS we used the acronym PECO, exposure - risk or prognostic factor. Population – patients from age 8–19 years with cleft lip and palate; Exposure - who underwent surgical treatment; Comparison - individuals of the same age without the condition; Outcomes - oral health-related quality of life assessed by specific psychometric scales designed for ORHQoL (COHIP: Child Oral Health Impact Profile; CPQ: Child Perceptions Questionnaires, and OHIP: Oral Health Impact Profile).

The included terms were related to “cleft lip and palate” and the predefined psychometric scales specifically designed for ORHQoL, adapted for use in other bibliographic databases, without the use of database filters. The PubMed term was as follows and adapted to other databases. Term: (“Cleft Lip“[MeSH] OR “Cleft Palate“[Mesh] OR “orofacial cleft”) AND (“Quality of Life“[MeSH] OR “quality of life” OR “QoL” OR “HRQL” OR “COHQOL” OR “HRQOL” OR “OHRQOL” OR “Oral Health-related Quality of Life” OR “COHIP” OR “Child Perception Questionnaire” OR “CPQ” OR “OHIP”). Search strategy for SciELO is closely related to the MEDLINE, as well as PsychNET, Scopus and Web Of Science. The search included articles from the inception of the aforementioned databases until August 2023, without language restriction. Grey literature was also considered; as well reference lists from included studies.

An a priori power analysis was conducted [24]. According to previous authors [25], we adopted an SMD of 0.30 as clinically relevant. In this sense, the adopted parameters were previously reported [24] to set the power analysis with an expected SMD of at least 0.30 (*d* = 0.30), with the minimum advised number of studies, plus a 20% margin (*k* = 12), a mean of at least 40 participants *per group* for each study arm (n_1_ = 40, n_2_ = 40), high heterogeneity (τ^2^) for a random-effects model and a significance level of p = 0.05. These parameters provided an effect power of 90.46%. For subgroup analyses to be performed, we set an intergroup SMD difference of at least two times the pre-estimated SMD and a maximum 7.5% for standard error for each group to meet a 80.74% power.

We included observational studies that presented a controlled, comparison or reference group, not necessarily blinded or randomized, and cross-sectional involving any form of non-syndromic CLP in patients aged 8–19 years, in which OHRQoL was assessed using scales designed to such aim or scales capable to identify aspects related to oral health instead of more general instruments of quality of life assessment. This aim was intended to meet for a more precise estimation of the patients’ quality of life given the conceptual nature of the constructs underlying the scales conception.

Exclusion criteria were studies that addressed CLP in genetic diseases and/or with reports of familial occurrence, maternal exposure to teratogens, unrelated groups for comparison purposes (patients vs. family members), studies that used general or unspecific QoL questionnaires, interviews, not validated inventories, incomplete reporting of scale data, or when it was not possible to extract data from the original study. Additionally, we excluded reviews, case reports, animal studies, computational models, and studies that used molecular approaches. The study selection was performed by two independent investigators (AGOJ and EM) with ties resolved by a third investigator (VZ).

The primary outcome was OHRQoL, with scores of subscales when possible. Other variables retrieved were sex, age, country and study type. No data imputing was performed.

A double screening for titles and/or abstracts of studies retrieved were independently performed by two review authors (AGOJ and EM) to identify studies that potentially meet the aims of the systematic review. The full text was retrieved and independently assessed by three authors (AGOJ, EM and VZ). Any disagreement over the eligibility was resolved through discussion with another collaborator (CPB). No translation was needed during the process, since no studies in languages other than English were retrieved. All steps were performed without any automated or machine learning processes.

### Data extraction

Data extraction was performed using an electronic form by two independent investigators (EM and VZ), with disagreements resolved by a third investigator (BB). No automation tools were used.

The data retrieved were author, date, study design, scale used for OHRQoL assessment, their scoring both overall and for domains when available, total and stratified sample in the study groups, considering sex, age and CLP classification. No data imputing was performed.

### Data synthesis

Studies considered eligible for synthesis were those containing data according to the eligibility criteria of patients and those that were possible to extract data, disregarding the primary study aim. No restriction to language or publication date was imposed, retrieving studies up to August, 2023.

The data was presented in a summary of evidence and synthesis as forest plots, with studies ordered by publication year.

A meta-analysis of continuous variables was performed using inverse variance for pooling estimates. Due to the inherent differences in scale scores we adopted a Standardized Mean Difference (SMD) as a summary measure. A random effects model was the first choice considering the expected between-study heterogeneity. We used the Hedges’ *g* for small-sample bias correction, the Paule-Mandel method to estimate the between-study variance (τ^2^) and its square root (τ), presented with 95% confidence intervals (95% CI) calculated by the method proposed by Jackson (2013). The Knapp-Hartung adjustments were used to calculate the confidence interval for the summary effect due to the expected variance for observational studies. Heterogeneity was estimated by the I^2^ [26] considering values of 50–75% as moderate, and values greater than 75% as high. The funnel and Baujat plots [27] were used to check for sources of heterogeneity and the Egger test for funnel plot asymmetry [28].

Sensitivity analyses were performed to verify possible interfering factors or variables of interest as follows: subgrouping based on the scale used in the study, removal of studies with discrepant effect sizes or risk of bias detected, and, finally, the analysis of domains of the scales [29]. Considering that subgroup analyses relies on the hypothesis that studies are not derived from a single population, the assumption is that each subgroup will present an overall effect, particularly considering variables with fixed levels such as age group an self-reporting assessment tools [24]. Thus, a common effect model was also adopted as a standpoint for effect comparison on the origin of potential differences in the observed effects, as well as mixed-effects model for subgroup analysis [24]. Analysis of the relationship between subgroups was performed using the χ^2^_2_ test with a mixed-effects model [24]. The results are presented as forest plots with SMD and 95% confidence intervals (CIs).

The scales used in these studies have the particularity of pointing their effects in opposite directions. The COHIP scale indicates a better OHRQoL when higher scores are observed. In contrast, the OHIP and CPQ scales work in the opposite direction, that is, lower scores indicate higher OHRQoL. Thus, for the last two scales it was necessary to multiply the extracted values by -1 to have estimated effects pointing to the same direction. Therefore, some values in forest plots appear to have a negative sign [30]. The scores were presented as means and standard deviations, and were transformed if reported otherwise, according to previous studies [31].

Meta-regression was performed using a mixed-effects model [32] considering the following variables: type of scale, scale domains and ROBINS-I assessment score. It was performed using the Paule-Mandel for τ^2^ estimator, the Knapp-Hartung method to calculate CI and *p* values, and included the intercept, which is the expected effect size for the Hedges’ *g* when the value of the predictor is zero. Results were presented as the estimate of the residual heterogeneity variance or the variance that is not explained by the predictor (τ^2^_unexplained_), the I^2^ equivalent, which is the variability that can be attributed to the remaining between-study heterogeneity after inclusion of the moderators, the R^2^ that indicates the difference in true effect sizes explained by the moderators of the meta-regression, the Test for Residual Heterogeneity, which is a Q-test to evaluate the significance of the heterogeneity not explained by the moderators, the Test of Moderators to check the influence of the predictors in the effect sizes of the studies, and, finally, the estimated regression coefficients, with 95% CI ranges, and all *p* values set at 0.05 for statistical significance [24].

Data analyses were performed with RStudio software (version 2022.02.2–485, The R Foundation for Statistical Computing, Vienna, Austria), using *meta* [32] and *dmetar* packages [33]. The PRISMA flow was produced with the online *app* [34].

### Risk of bias assessment

The risk of bias assessment was performed by two independent investigators (AGOJ and EM), with disagreements resolved by a third part (VZ). We used the ROBINS-I tool for risk of bias assessment, and the results are presented as charts according to the defined by the authors [35]. Certainty assessment is reported as 95% CI without previous definition of the limits considering that the spectrum and the approach to CLP patients are performed under well-established settings of clinical practice guidelines [10].

## Results

### Systematic review and summary of evidence

**Fig. 1:**
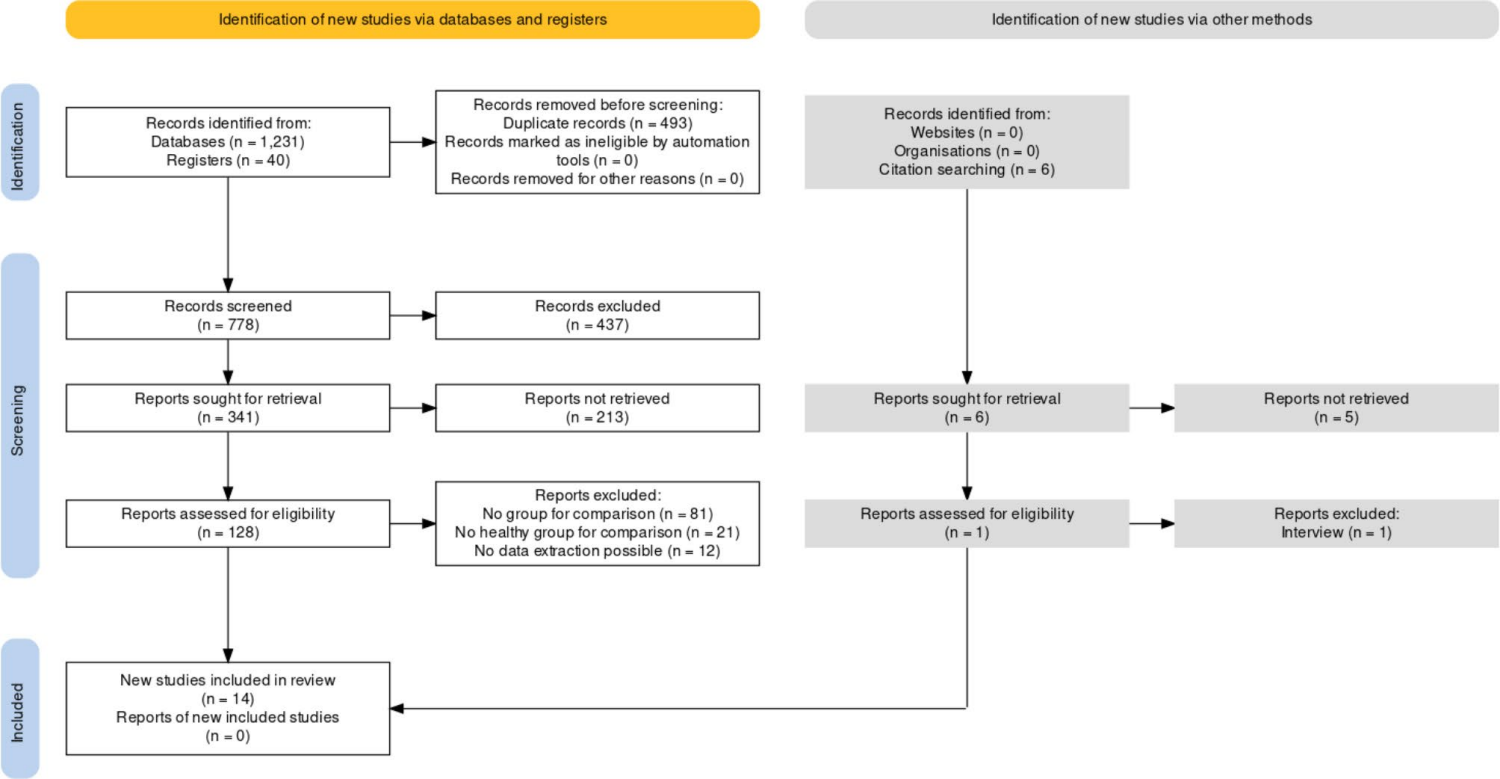
PRISMA flowchart of study selection

Fourteen studies were included in this meta-analysis, comprising 1,185 children and adolescents with CLP and 1,558 healthy controls. Figure 1 presents a PRISMA flowchart of the studies included in the systematic review. A summary of this evidence is presented Table 1 [14, 36–48].

**Table 1:** Summary of evidence of the studies included

Author	Year	Country	N		Mean Year (yo)^a^			Sex (N)		OHRQoL Scale	Domains	Case^b^	Control^b^
			Case	Control	Case		Control						
Locker [36]	2005	Canada	39	32	12.2 ± 1.5		12.2 ± 1.4			CPQ_11 − 14_	Overall	31.40 ± 3.40	23.20 ± 2.2
								F	30		Oral symptoms	7.40 ± 1.3	7.20 ± 0.9
								M	41		Functional limitations	8.10 ± 2.1	5.20 ± 0.4
											Emotional well-being	7.20 ± 2.2	6.30 ± 1.8
											Social well-being	8.80 ± 1.8	4.70 ± 1.5
Wogelius [37]	2009	Denmark	15	97	8–10		8–10	F	NA	CPQ_8 − 10_	Overall	7.90 ± 8.0	8.50 ± 6.2
			21	154	11–14		11–14	M	NA	CPQ_11 − 14_	Overall	10.2 ± 7.2	10.5 ± 7.6
Ward [38]	2013	USA	75	75	8–14		8–14			COHIP	Overall	95.6 ± 18.3	108.6 ± 16.9
											Oral health well-being	24.8 ± 5.9	26.9 ± 6.5
								F_case	27		Functional well-being	17.7 ± 4.7	20.9 ± 3.6
								M_case	48		Social-Emotional well-being	24.3 ± 6.7	27.7 ± 6.1
								F_control	46		School Environment	13.1 ± 3.0	14.7 ± 2.0
								M_control	29		Self-Image	15.7 ± 4.3	18.4 ± 4.9
											Treatment Expectancy	5.1 ± 1.4	5 ± 1.4
											Global Health	3.1 ± 0.8	3.1 ± 0.9
Antoun [39]	2015	New Zealand	24	30	12.6 ± 2.8		14.5 ± 1.9			OHIP 14	Overall	10.5 ± 10.8	11.6 ± 10.9
								F_case	10		Functional Limitations	1.75 ± 1.7	1.23 ± 1.5
								M_case	14		Physical Pain	1.25 ± 1.7	1.5 ± 1.8
								F_control	13		Psychological Discomfort	2.54 ± 2.3	3 ± 2.5
								M_control	17		Physical Disability	0.75 ± 1.3	0.73 ± 1.6
											Psychological Disability	1.92 ± 2.1	2.67 ± 2.4
											Social Disability	1.17 ± 1.7	1.27 ± 2.1
											Handicap	1.1 ± 1.4	1.2 ± 1.6
Kragt [40]	2016	Netherlands	30	213	10–14		10–14	F	122	COHIP 38	Overall	159 ± 17.5	167.26 ± 13.3
								M	121				
Long [41]	2016	USA	527	362	12.6 ± 3.1		11.5 ± 2.9	F	391	COHIP	Overall	96.2 ± 18.8	100.1 ± 17.7
								M	498				
Kortelainen [42]	2016	Finland	26	71	11–14		11–14	F_case	13	CPQ_11 − 14_	Overall	55.5 ± 12.3	15.0 ± 11.0
								M_case	13		Oral symptoms	11.9 ± 3.5	5.1 ± 2.7
								F_control	31		Functional limitations	14 ± 5.0	2.8 ± 2.7
								M_control	40		Emotional well-being	12.6 ± 3.3	4.2 ± 5.2
											Social well-being	17.1 ± 4.6	2.9 ± 3.5
Aravena [12]	2017	Chile	48	96	11.3 ± 2.3		11.2 ± 2.3			COHIP-SP	Overall	94.1 ± 19.3	97.1 ± 15.6
								F_case	18		Oral health well-being	24.5 ± 6.0	25.1 ± 5.6
								M_case	30		Functional well-being	16.8 ± 4.2	19.2 ± 4.1
								F_control	46		Social-Emotional well-being	22.5 ± 7.4	24.4 ± 5.6
								M_control	45		School Environment	12.9 ± 2.6	14.3 ± 2.2
											Self-Image	17.2 ± 5.3	13.9 ± 4.6
Boy-Lefèvre [43]	2018	France	45	92	NA		NA			CPQ_8 − 10_	Overall	17.73 ± 11.2	11.83 ± 11.1
								F	NA		Oral symptoms	4.41 ± 2.9	4.63 ± 3.9
								M	NA		Functional limitations	3.95 ± 3.6	2.35 ± 3.3
											Emotional well-being	2.95 ± 4.0	2.51 ± 3.5
											Social well-being	6.41 ± 4.6	2.34 ± 3.4
Nichols [44]	2018	New Zealand	19	16	12.7 ± 3.1		15.1 ± 2.1			OHIP 14	Overall	12.4 ± 11.4	11.2 ± 11.0
											Functional Limitations	1.3 ± 1.5	1.9 ± 1.8
								F_case	7		Physical Pain	1.4 ± 1.5	1.4 ± 1.8
								M_case	12		Psychological Discomfort	3.4 ± 2.7	2.6 ± 2.3
								F_control	9		Physical Disability	0.7 ± 1.4	0.8 ± 1.3
								M_control	7		Psychological Disability	2.6 ± 2.6	2.1 ± 2.2
											Social Disability	1.6 ± 2.4	1.3 ± 1.9
											Handicap	1.4 ± 1.8	1.2 ± 1.4
Montes [45]	2019	Brazil	54	54	9.07 ± 0.8		9.07 ± 0.8	F_case	23	CPQ_8 − 10_	Overall	17.2 ± 13.0	13.4 ± 12.1
								M_case	31		Oral symptoms	6 ± 2.8	5.2 ± 3.8
								F_control	23		Functional_limitations	3.7 ± 3.5	2.4 ± 3.8
								M_control	31		Emotional well-being	3.1 ± 3.7	3.2 ± 3.7
											Social well-being	4.4 ± 5.1	2.7 ± 4.2
Nagappan [46]	2019	India	80	80	8–16	8–16				COHIP 25	Overall	70.6 ± 14.1	111.82 ± 12.3
											Oral health well-being	14.9 ± 7.9	31.3 ± 3.2
								F_case	29		Functional well-being	7.6 ± 2.7	19.3 ± 6.2
								M_case	51		Social-Emotional well-being	21.3 ± 8.1	26.2 ± 5.4
								F_control	36		School Environment	12.1 ± 2.1	14.22 ± 0.9
								M_control	44		Self-Image	14.7 ± 3.9	20.8 ± 0.9
											Treatment Expectancy	4.2 ± 1.4	5.1 ± 0.02
											Global Health	2.9 ± 0.6	3.5 ± 1.1
Francisco [47]	2021	Portugal	111	115	8–27	8–27				OHIP 14	Overall	10.2 ± 7.2	9.4±
											Functional Limitations	1.6 ± 1.4	1.3±
								F_case	45		Physical Pain	3 ± 1.6	3.2±
								M_case	66		Psychological Discomfort	1.5 ± 1.9	1.3±
								F_control	45		Physical Disability	1.3 ± 1.6	1.5±
								M_control	66		Psychological Disability	1.1 ± 1.6	1±
											Social Disability	1.4 ± 1.5	0.8±
											Handicap	0.3 ± 0.9	0.3±
Defabianis [48]a	2022	Italy	32	32	8–11					COHIP	Overall	101.2 ± 15.1	119.5 ± 7.5
											Oral health well-being	27 ± 4.5	32.2 ± 3.2
								F_case	31		Functional well-being	17.3 ± 3.7	21.6 ± 1.9
								M_case	40		Social-Emotional well-being	26 ± 7.2	31 ± 1.8
								F_control	31		School Environment	12.7 ± 2.2	15.3 ± 1
								M_control	40		Self-Image	18.1 ± 5	19.4 ± 2.7
Defabianis [48]b			39	39	12–18					COHIP	Overall	92.5 ± 19.3	116 ± 11.9
											Oral health well-being	26.8 ± 5.7	31.8 ± 4.9
											Functional well-being	18 ± 3.3	21.7 ± 2.5
											Social-Emotional well-being	22.1 ± 8.4	29.3 ± 4.2
											School Environment	12.2 ± 3	14.9 ± 1.8
											Self-Image	13.3 ± 4.8	18.4 ± 3.2

^a^Age was presented as mean and standard deviation (± sd) and, when not available, the age range considered in the study. ^b^Score of the scales and respective domains was presented as mean and standard deviation (± sd). COHIP: *Child Oral Health Impact Profile;* CPQ: *Child Perceptions Questionnaires;* F: Female; M: Male; NA: Not presented; OHIP: *Oral Health Impact Profile;* yo: years old

According to the predefined power analysis, the present study surpassed the estimated and reached statistical power for both the meta-analysis and subsequent subgroup analyzes (*k =* 14, SMD = 0.45 and mean participants *per study* arm, n = 50).

### Meta-analysis

**Fig. 2:**
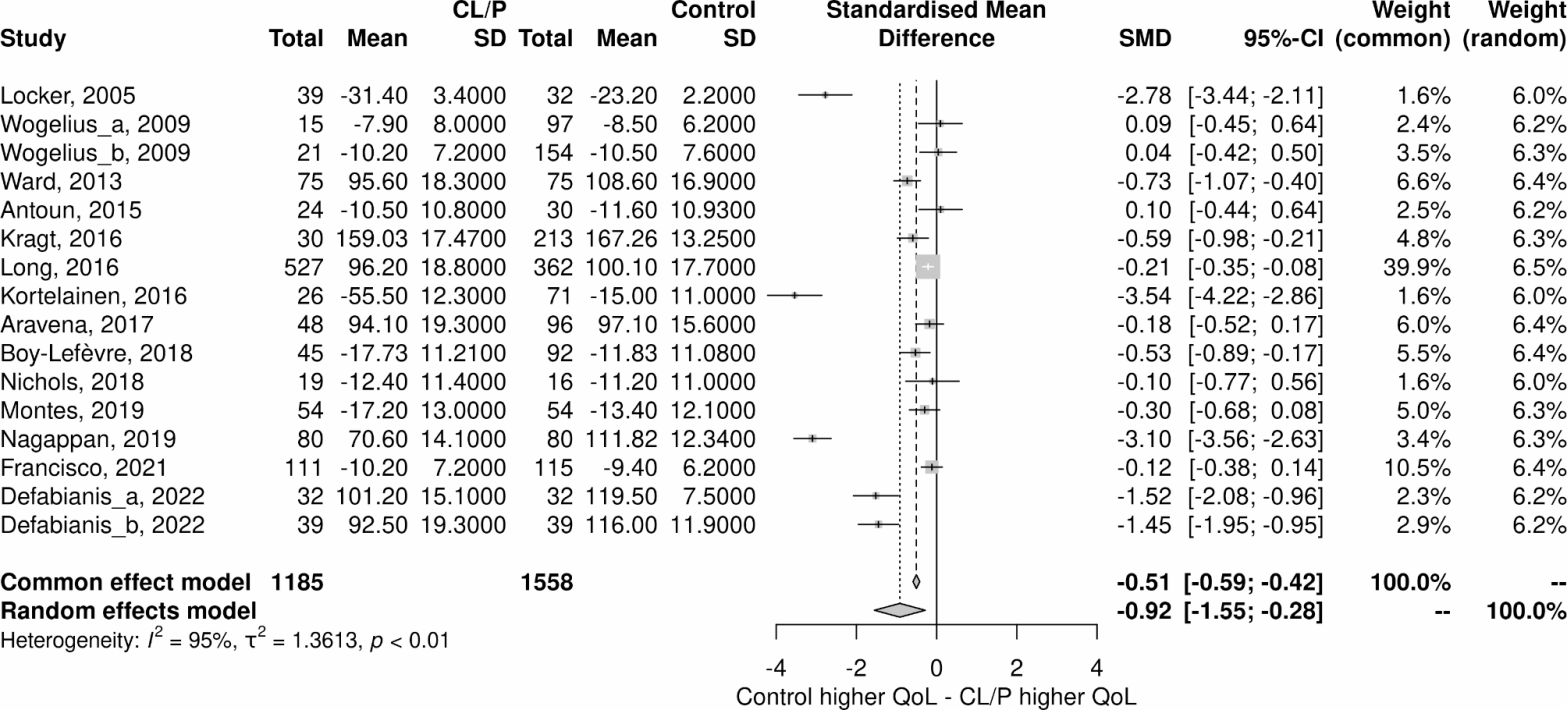
Forest plot of the studies included

As shown in Fig. 2, the OHRQoL was higher in the Control group than in the CLP group. (-0.92; 95% CI:-1.55;-0.28). The heterogeneity was I^2^ = 95%.

**Fig. 3:**
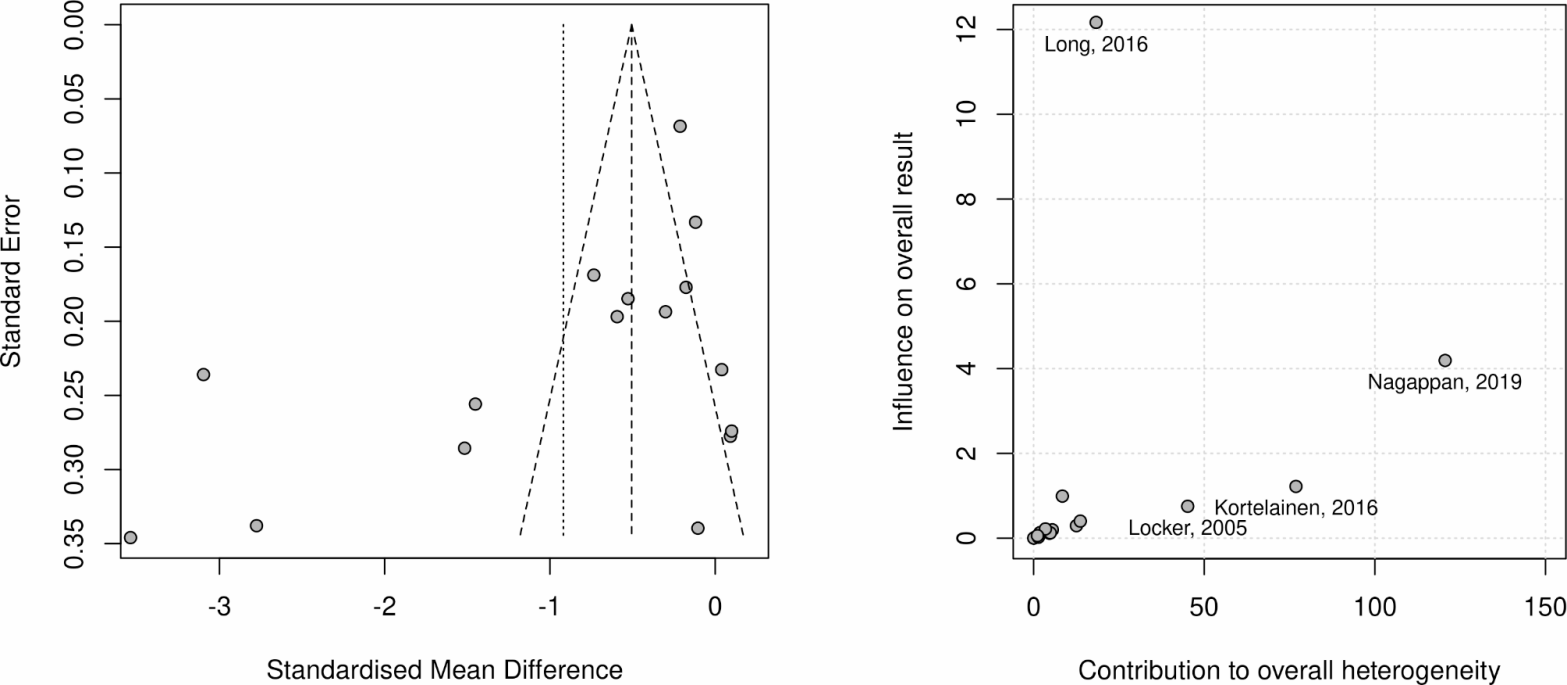
Risk of bias analysis: Funnel plot (**A**) and Baujat plot (**B**)

Figure 3 presents the funnel and Baujat plots for the risk of bias analysis. The funnel plot shows strong asymmetry, and the Baujat plot indicated three studies with a strong influence on overall heterogeneity, which were removed for the sensitivity analysis.

### Meta-regression

The meta-regression was performed for each variable at once instead of a multiple regression. Considering the scale type, we found τ^2^_unexplained_ = 0.8679 (SE = 0.4703), I^2^ equivalent = 95.65%, R^2^ = 0.12%, Test for Residual Heterogeneity (p < 0.001), Test of Moderators (p = 0.0375), and the estimated regression coefficients for each scale, being COHIP (p = 0.0286, 95%CI [-2.0573–0.1336]), CPQ (p = 0.0384, 95%CI [-2.1799–0.0699]) and OHIP (p = 0.9523, 95%CI [-1.5309–1.4469]).

**Fig. 4:**
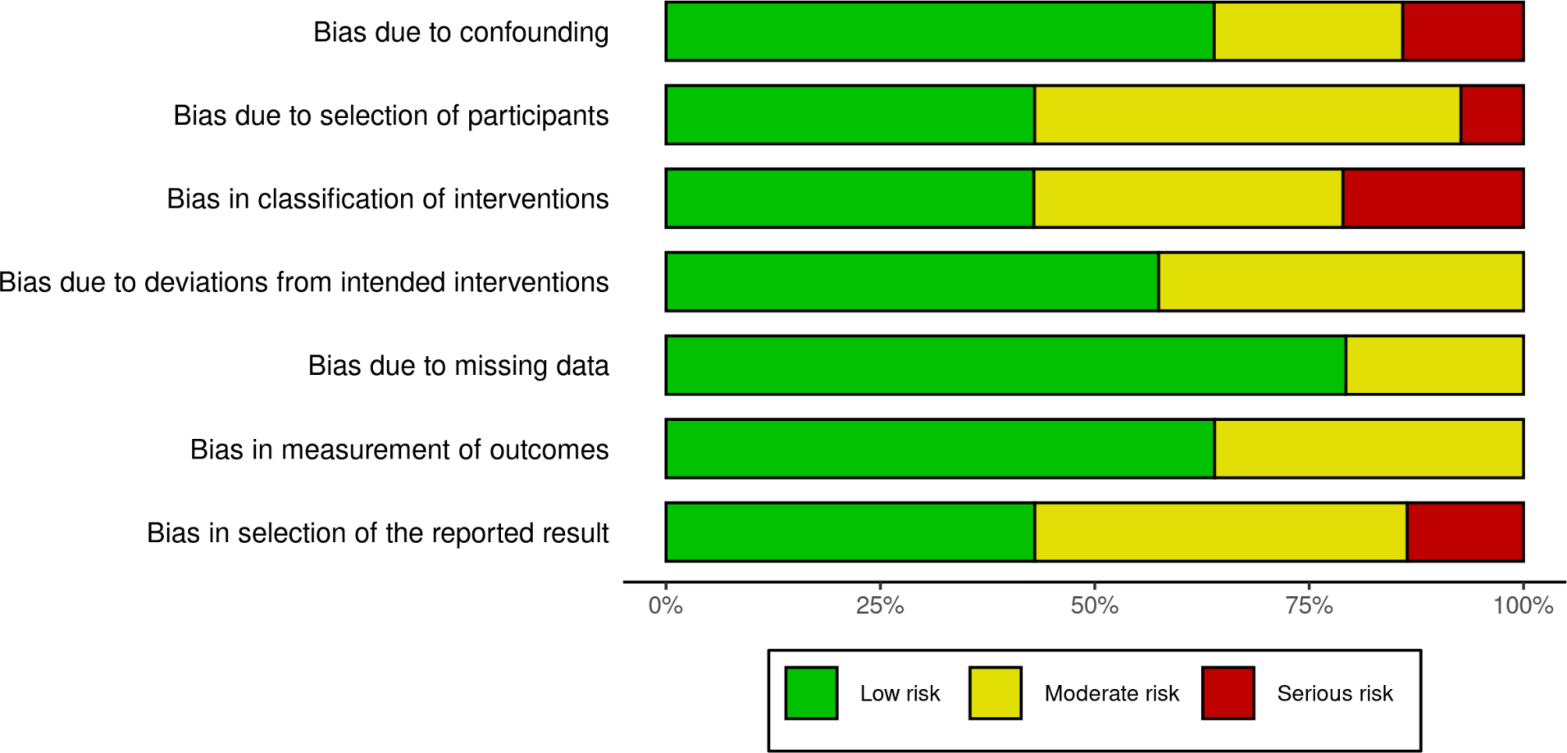
Risk of bias by overall risk level

Considering the ROBINS-I classification, we found τ^2^_unexplained_ = 1.2438 (SE = 0.5106), I^2^ equivalent = 97.04%, R^2^ = 6.85%, Test for Residual Heterogeneity (p < 0.0001), Test of Moderators (p = 0.2440), and the estimated regression coefficients for each category, being Low risk (p = 0.3137, 95%CI [-1.7402 – -0.2183]), Moderate risk (p = 0.5348, 95%CI [-0.9945–1.8274]) and Serious risk (p = 0.0995, 95%CI [-0.3013–3.0648]) (Fig. 4).

**Fig. 5:**
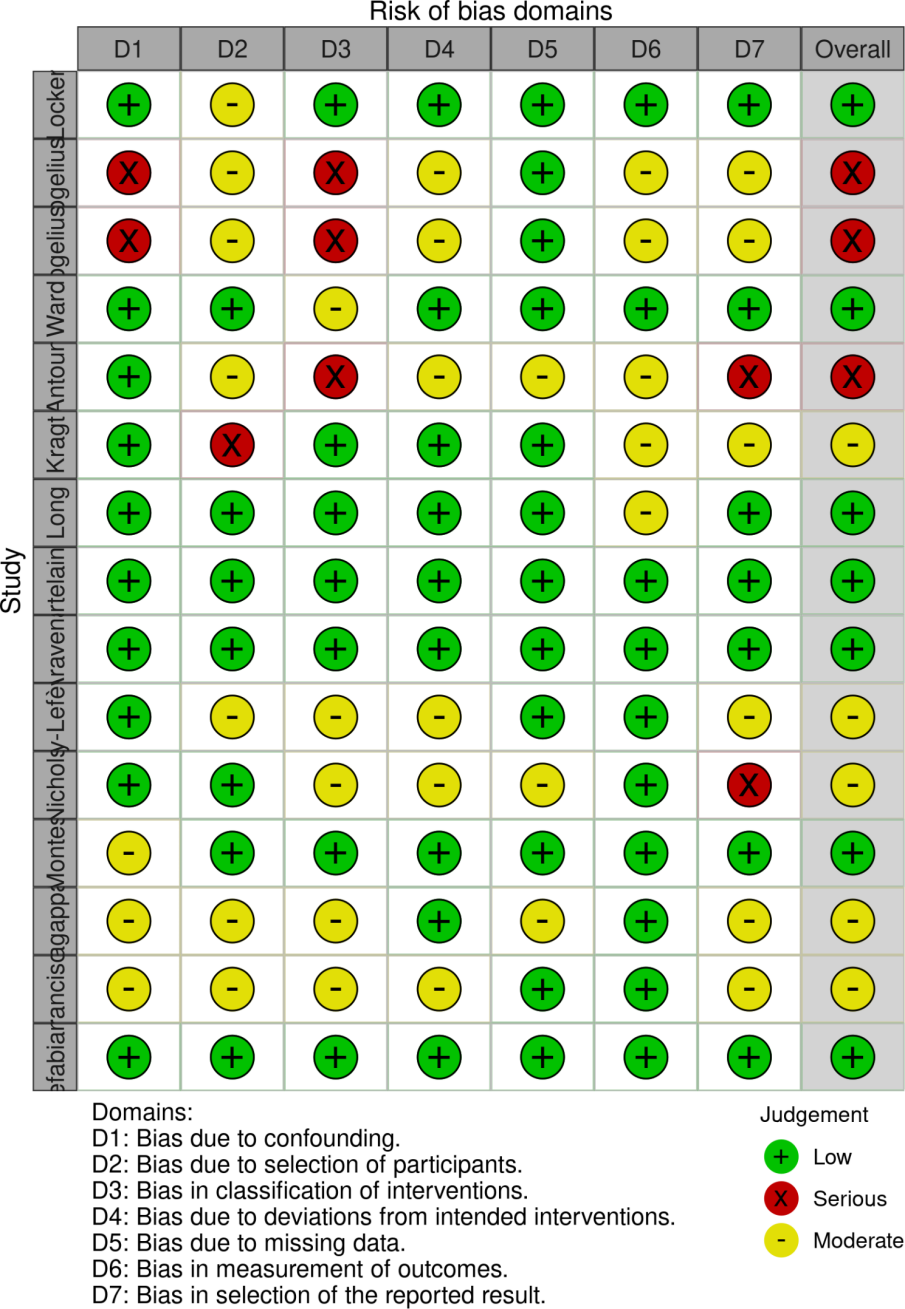
Risk of bias by domains

Considering the scale Domains, we found τ^2^_unexplained_ = 0.7710 (SE = 0.2097), I^2^ equivalent = 94.82%, R^2^ = 0.00%, Test for Residual Heterogeneity (p < 0.001), Test of Moderators (p = 0.0001), and the estimated regression coefficients for each category, being Emotional domain (p = 0.0483, 95%CI [-1.1681 – -0.0046]), Functional domain (p = 0.0004, 95%CI [-1.5699 – -0.4972]), and Social domain (p = 0.0003, 95%CI [-1.6133 – -0.5397]) (Fig. 5).

### Sensitivity analysis

**Fig. 6:**
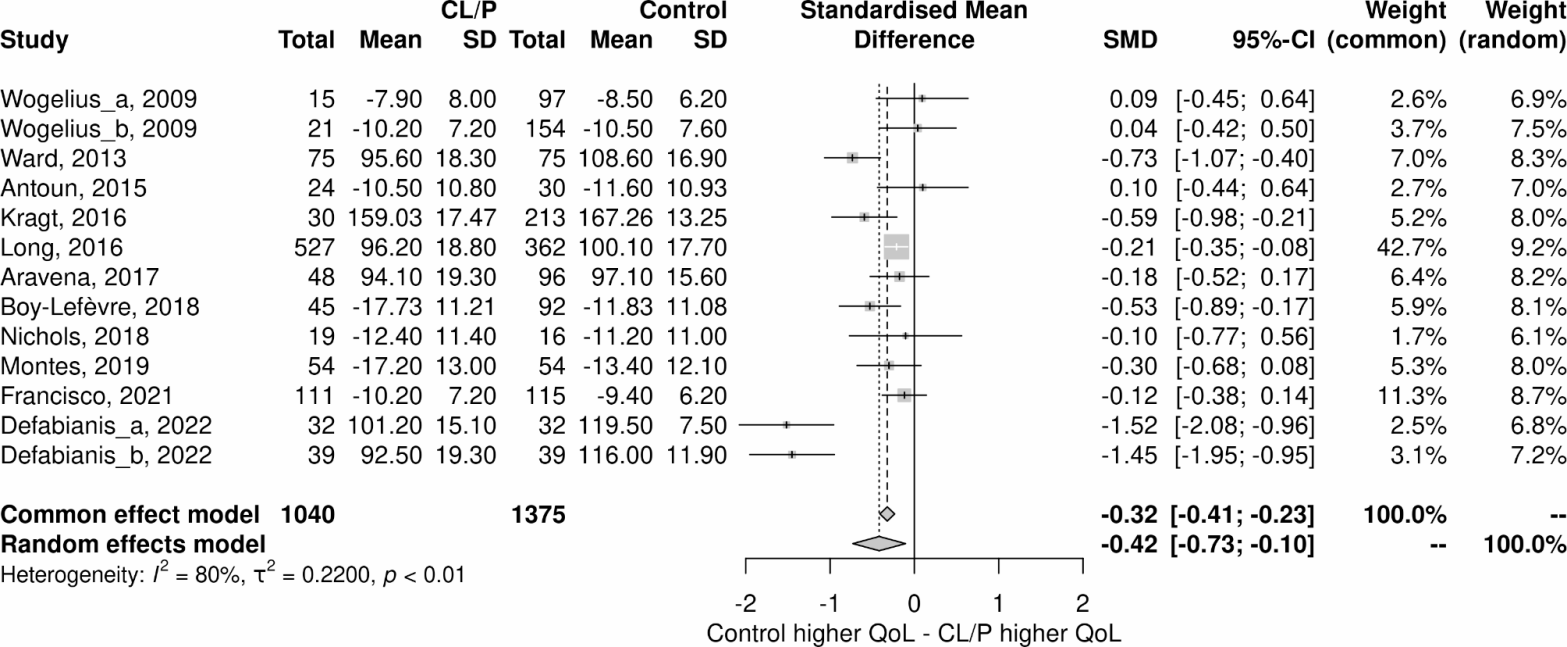
Forest plot with the global results of the scales used to assess oral health-related quality of life after removing discrepant studies

Sensitivity analyses were performed by removing studies that presented the most extreme deviations in effect sizes [36, 42, 46] (Fig. 6). The results changed substantially with a significant reduction in the difference between the original SMD values and after the exclusion of studies for both the case and control groups (-0.42; 95% CI, -0.73 – -0.10), with closer values for fixed effects and random effects, and lower heterogeneity (I^2^ = 80%).

**Fig. 7:**
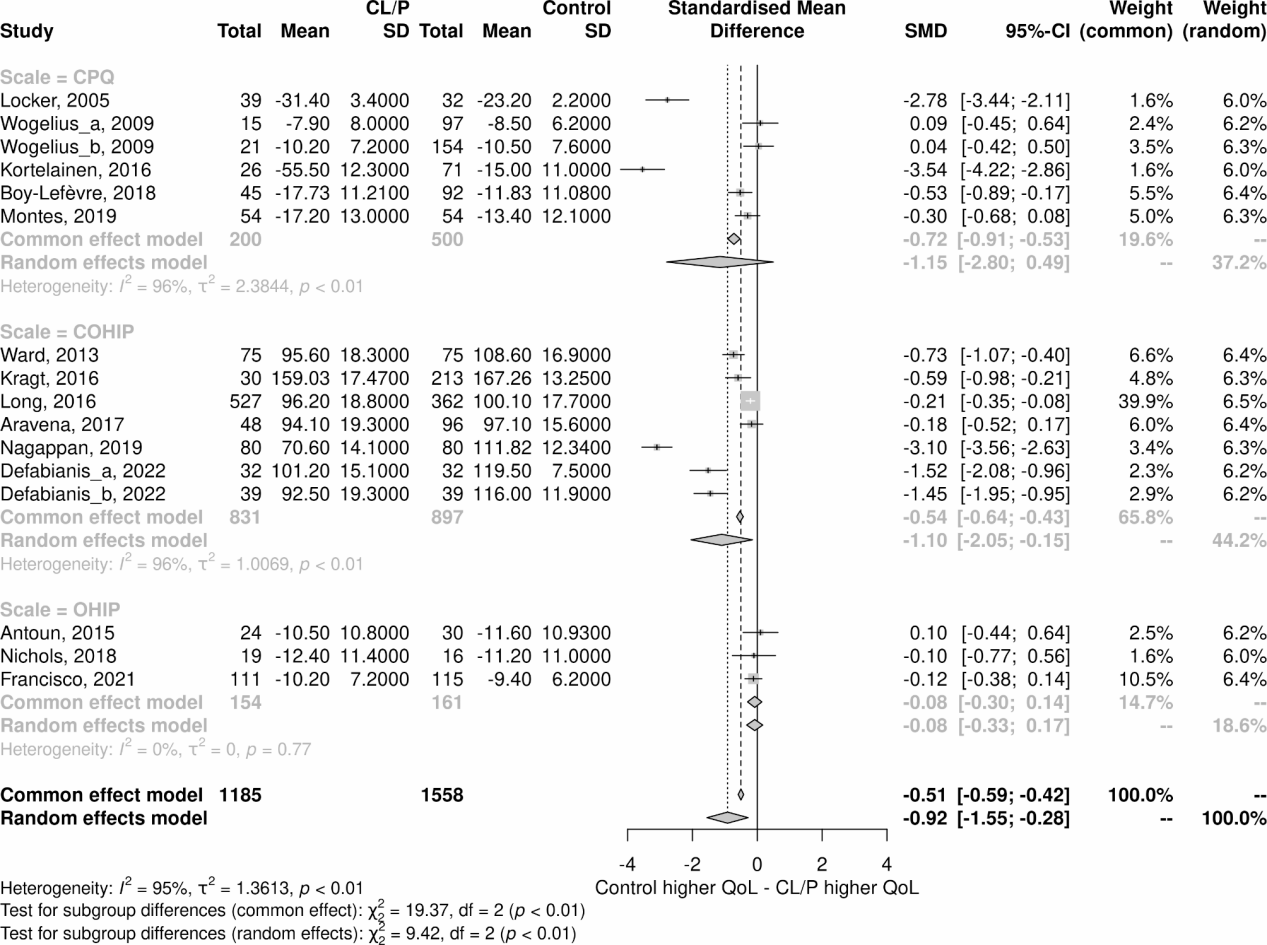
Subgroup analysis considering the different scales to assess oral health-related quality of life

In the analysis of subgroups considering the scales used to assess QoL related to oral health, it was decided to preserve discrepant studies as a means of identifying possible relationships with these subgroups. Thus, the subgroup analysis indicated that the CPQ and COHIP scales presented very discrepant SMD values, despite pointing to the same effect direction. In contrast, the OHIP scale showed a non-significant difference between cases and controls, with estimates much lower than the other two scales (Fig. 7).

**Fig. 8:**
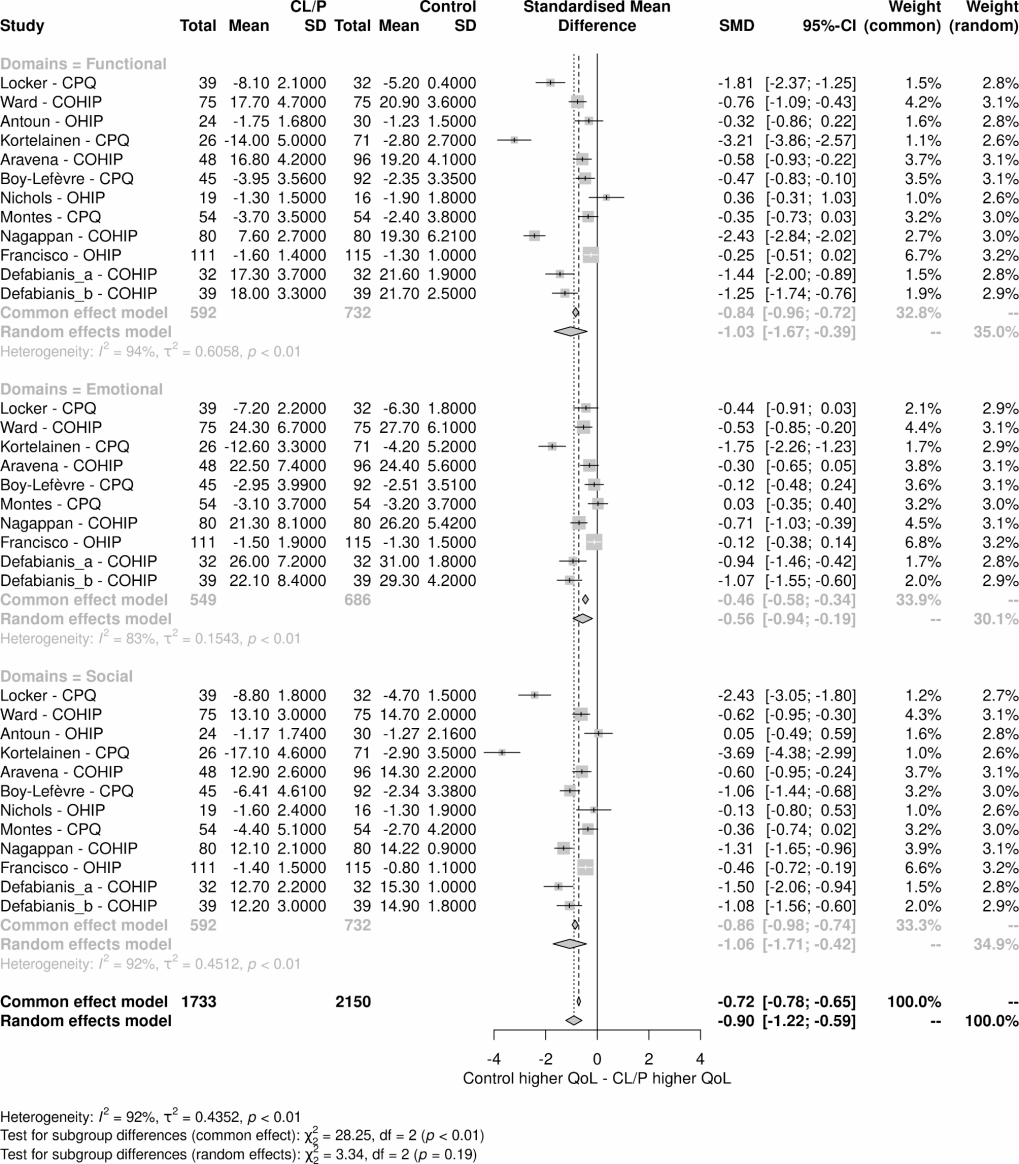
Subgroup analysis considering domains of the scales used to assess oral health-related quality of life

Finally, the domains of the scales whose constructs were more associated with general QoL were evaluated, which could indicate which variables measured by the scales may contribute to the overall result observed. Among the studies that presented data, domains that assessed the aspects of oral functionality, emotional well-being, and social well-being were selected (Fig. 8).

These results suggest that OHRQoL associated with oral functionality and social well-being is more influential on outcomes than emotional well-being. The results for these domains were higher than the overall result, both in the analysis of the general score and the score of the selected domains. However, the result for emotional well-being presents values close to half of the global value, indicating that it is a less influential aspect of the outcomes assessed by the scales.

## Discussion

This study assessed the OHRQoL outcomes of children and adolescents with CLP through a systematic review and meta-analysis. To date, this is the first meta-analysis on QoL in CLP that exclusively considers scales developed to assess OHRQoL and that have the discriminant capacity to assess individuals with CLP. In the global analysis, the results showed that QoL was slightly better in the control group.

It is acknowledged that the repair of CLP requires many surgical interventions, accompanied by several long-term treatments for occlusion, mastication, speech, and facial appearance. The burden of comprehensive care is widely recognized, as is the need for a multidisciplinary approach to managing and supporting patients and their families. Therefore, it was initially expected that the control group would have better QoL indices. In contrast, the small difference found here seems to be consistent with results from other studies that found even more unexpected results in CLP patients with better OHRQoL [49]. Among the many possible explanations, it can be conjectured that a patient suffering from the condition does not know a different life than that as a carrier of the condition. In addition, in wealthier countries, the most extensive surgical interventions take place in the first years of life and therefore limit the impact on the patient’s life [22].

QoL is a latent trait affected by numerous variables, even when limiting the analysis to health-related and OHRQoL, or when focusing on specific aspects of the patients’ condition. OHRQoL is an indicator of oral health that is commonly used to assess the functional, emotional, and psychosocial impacts of oral diseases and disorders [36]. Although socio-dental indicators developed for adults have been successfully applied to adolescents, the perception of adults and children regarding the impact of health problems on QoL is different, since children and adolescents have a peculiar view of themselves and the world owing to their stage of physical and emotional development. In addition, confounding factors may change according to stages of development. For instance, anxiety, stress and discomfort with perioperative and postoperative procedures are acknowledged causes of impairment in QoL. However, it is not constant in the lifespan, as well as it is different for people that undergo regular medical appointments in contrast to those that are not in such condition. Moreover, orofacial cleft isolated or occurring with another condition/syndrome, and low educational attainment can have along-lasting adverse impact on social, mental and physical health outcomes [50–52]. Previous studies indicated that confounding factors that represent a major impact to non-CLP patients’ quality of life not necessarily have the same effect on CLP patients [51–54]. Furthermore, studies also showed that the QoL measured by non-specific instruments are even more conflicting and present strong ceiling/floor effect, which means that general quality of life scales do not reach certain limits or do not detect some specificities. This led to the creation of more specific instruments and a narrower approach [51, 52, 54]. Therefore, the development of specific instruments for children and adolescents would enable a more accurate measurement of the impact of oral problems on QoL.

The assessment of OHRQoL involves a complex conceptual framework and methodological issues in the construction of self-report indicators of children’s health status. The structure of children’s self-concept and perception of health is age-dependent because of their ongoing cognitive, emotional, social, and language development. Likewise, the content of daily activities, understanding of feeling states, perception of relationships, and communication skills evolve with age. These age differences in cognitive, emotional, functional, and behavioral characteristics must be accommodated in a child health status questionnaire [55]. Considering this, Jokovic et al. 2002 [55] and 2004 [56] constructed the CPQ and Broder et al. (2007) [57] developed the COHIP.

There are two versions of the CPQ, one for children aged 8–10 years and for children aged 11–14 years. The authors claim that differences in development, self-perception, and socialization are critical for understanding the condition [55]. In contrast, no continuity between the two scales is criticized, and their use in cohort studies is limited [58]. Thus, the COHIP, a version for children and adolescents aged between 8 and 15 years [57] derived from the OHIP for adolescents and adults was developed and later adapted for up to 17 years [58], which has also been used in patients up to 19 years of age. This study aimed to produce a scale that considers the ability to understand and communicate beyond the recognized particularities of each stage of development [59]. Despite the existence of other scales developed recently, the amount of data and the diversity of experimental designs adopted are still emerging in the literature.

In the present study, the analysis of subgroups considering the scales used to assess OHRQoL indicated that the CPQ and COHIP scales presented higher effect sizes and heterogeneity, despite pointing to the same effect direction. In contrast, the OHIP scale showed a non-significant difference between cases and controls, with estimates much lower than the other two scales.

The subgroup test also showed differences among groups under the common effect, but not in the random effects model. This observation suggests that, despite the expected differences among different samples, there are no differences in terms of the OHRQoL outcomes measured by the different scales. This finding is not absolutely surprising since it’s widely acknowledged that facial deformities may vary in intensity, but not in the individuals’ self-perception [60]. In this case, the intensity and gravity of the CLP may be not so relevant for the patient as the deformity itself. In other words, even a discrete or mild deformity is capable to cause impairments in QoL at the comparable extent to a bilateral or even more complex CLP. In this case, our data suggest that the multiprofessional health support team should consider every patient as potentially affected in terms of OHRQoL impairment with the same or much closer intensity.

The main consequences of CLP are facial and functional impairments, which appear to be closely related to difficulties in psychosocial functioning. Several studies have considered satisfaction with facial appearance when assessing QoL [15, 59] and support that this feature plays an important role in predicting psychosocial adjustment [61]. In terms of function, speech impairment is considered the main problem and can influence the social lives of people with CLP [15, 58, 62]. These data are corroborated by the findings of the present study regarding the influence of oral functionality and social well-being domains on the overall result. In contrast, emotional well-being has a lower negative impact on the observed general QoL, which may be the result of psychosocial interventions as well as the individual’s adjustment to their condition.

In the present study, some domains of the scales whose constructs were more associated with general QoL, which could indicate which variables measured by the scales could contribute to the overall result, were also evaluated. The results suggest that the OHRQoL associated with oral functionality and social well-being is more influential on the results than emotional well-being.

The meta-regression showed that none of the R^2^ indicates that the difference in true effect sizes can be explained by the moderators. The Test for Residual Heterogeneity with p-values < 0.05 indicates that the heterogeneity not explained by the predictor is significant, suggesting that there are other factor affecting the heterogeneity. The Test of Moderators with p-values < 0.05 indicates that the predictor influence the effect size of the studies, suggesting that the moderators selected may be relevant for part of the results observed. Regarding the estimated regression coefficients, none of the scales showed significant p-values, suggesting that the scales are not significant for the differences observed. In other words, they may be comparable in terms of the precision in the assessment of the OHRQoL or they share a common systematic error. Despite the speculative nature of this point, it may be considered a form of equivalence in absolute terms advising for the adoption of one of the scales at once instead of mixing them since they are not comparable in scoring. Regarding the risk of bias, studies with Low risk presented significant p-values, suggesting that lower risks may indicate more accurate assessments. Finally, regarding the domains of the scales, it is interesting that the functional and social domains have statistically significant regression estimates, but not the emotional domain. This suggests that the CLP patients are challenged by functional issues, perhaps associated with the burden of the long treatment and several interventions, social issues, possibly associated with facial deformities and the consequences of sociability, but not with emotional domain, suggesting that the aforementioned adjustment to the condition may have a significant adaptive role for those affected by the condition.

This study presents limitations. The COHIP, OHIP, and CPQ_11 − 14_ are self-applied scales, and the CPQ_8 − 10_ scale can be administered by caregivers or health care professionals. The age group of the participants in the studies included in this meta-analysis ranged from 8 to 19 years as well as the age group validated for the scales, which may interfere in the different perceptions of QoL in childhood and adolescence, in addition to the different stages of the treatment. Furthermore, no distinction in terms of CLP severity or sex was possible, being a potential source of bias. Additionally, the influence of factors such as socioeconomic status and other demographic characteristics received limited attention in most of the studies included, and the differences owing to these factors on OHRQoL may have been underestimated.

Despite these limitations, the study strengths rely on the fact that the results of the meta-analysis for the fixed effect model were homogeneous in the analyzes, indicating that despite the enormous variety of clinical conditions and other variables, the presence of CLP can be a less heterogeneous condition from the QoL standpoint and other psychosocial aspects compared to the broad spectrum of clinical manifestations of the condition. This finding may induce the adoption of more inclusive approaches for multidisciplinary teams, facilitating the detection and prioritization of individual patient issues and demands. Therefore, outcomes can be monitored by considering different responses to treatment. Another point to be highlighted is the already recognized importance of early intervention in patients with CLP, in addition to the functional and esthetic results, which have already been widely studied. The small difference in QoL measured in favor of the healthy group, mainly due to functional and social issues, reinforces the notion that work involving emotional well-being has been successful. This finding suggests that the impact of the condition on QoL can be even more attenuated when considering social aspects in a multidisciplinary healthcare approach. Thus, more than the analysis of generic QoL instruments or comparisons of psychometric properties of available scales, we presented data from the sample population of interest for diagnostic accuracy on widely used scales. Additionally, this is the first meta-analysis to assess the results of scales specifically designed for OHRQoL that can also be used in CLP patients to improve the understanding of the complex trait of QoL in patients with CLP. Finally, our data provide further guidance for policymakers, researchers, and health professionals in evaluating health interventions and prioritizing the allocation of health resources.

## Conclusions

In conclusion, the global OHRQoL is slightly worst in the CLP patients than control group. The difference between OHRQoL was mainly detected through OHIP. The most affected domains are functional, emotional and social, indicating that more than emotional support, the multidisciplinary healthcare team may provide further attention to aspects related to functional and social issues related to the OHRQoL in CLP patients.

## Data Availability

The datasets generated and/or analysed during the current study are available from the corresponding author.

## List of abbreviations

CI: Confidence intervals
CLP: Cleft lip and palate
COHIP: Child Oral Health Impact Profile
CPQ: Child Perceptions Questionnaire
NOS: Newcastle-Ottawa scale
OHIP: Oral Health Impact Profile
OHRQoL: Oral health-related QoL
PRISMA: Preferred Reporting Items for Systematic Reviews and Meta-Analysis
QoL: Quality of life
SMD: Standardized mean difference
